# Wine yeast phenomics: A standardized fermentation method for assessing quantitative traits of *Saccharomyces cerevisiae* strains in enological conditions

**DOI:** 10.1371/journal.pone.0190094

**Published:** 2018-01-19

**Authors:** Emilien Peltier, Margaux Bernard, Marine Trujillo, Duyên Prodhomme, Jean-Christophe Barbe, Yves Gibon, Philippe Marullo

**Affiliations:** 1 Univ. Bordeaux, ISVV, Unité de recherche OEnologie EA 4577, USC 1366 INRA, Bordeaux INP, Villenave d’Ornon, France; 2 Biolaffort, Bordeaux, France; 3 Pernod Ricard, Creteil, France; 4 INRA, University of Bordeaux, UMR 1332 Fruit Biology and Pathology, Villenave d’Ornon, France; University of Strasbourg, FRANCE

## Abstract

This work describes the set up of a small scale fermentation methodology for measuring quantitative traits of hundreds of samples in an enological context. By using standardized screw cap vessels, the alcoholic fermentation kinetics of *Saccharomyces cerevisiae* strains were measured by following their weight loss over the time. This dispositive was coupled with robotized enzymatic assays for measuring metabolites of enological interest in natural grape juices. Despite the small volume used, kinetic parameters and fermentation end products measured are similar with those observed in larger scale vats. The vessel used also offers the possibility to assay 32 volatiles compounds using a headspace solid-phase micro-extraction coupled to gas chromatography and mass spectrometry. The vessel shaking applied strongly impacted most of the phenotypes investigated due to oxygen transfer occuring in the first hours of the alcoholic fermentation. The impact of grape must and micro-oxygenation was investigated illustrating some relevant genetic x environmental interactions. By phenotyping a wide panel of commercial wine starters in five grape juices, broad phenotypic correlations between kinetics and metabolic end products were evidentiated. Moreover, a multivariate analysis illustrates that some grape musts are more able than others to discriminate commercial strains since some are less robust to environmental changes.

## Introduction

In the last decade, the emergence of high throughput sequencing has opened perspectives for studying the genetic adaptation of microbial species in their specific environments [1]. This is the case for the wine related microbes found in ecological niches that continuously change and from grape must to wine [2,3]. Thanks to the reduction in the genome sequencing costs, large comparative genomic studies were carried out at the intraspecific level for lactic bacteria (*Oenococcus oeni*) [4] and various yeast species, including *Saccharomyces uvarum* [2,3], *Bretanomyces bruxellensis* [5,6] and *Saccharomyces cerevisiae* [7]. The bioinformatics analysis of such genomes shed light on genomic adaptation mechanisms such as chromosomal introgression [5], chromosomal translocations [8,9], horizontal transfer [10,11], polyploidy [5,6]; for an extensive review see [12]. Population genetics studies clearly demonstrates that the wine microbes show domestication signatures compared to other strains isolated in different biotopes [4,5,13–15].

To bridge the gap existing between this overall diversity and the specific molecular mechanisms of phenotypic adaptation, quantitative genetics approaches such Quantitative Trait Loci (QTL) mapping or Genome Wide Association Studies (GWAS) are usually used [16]. QTL mapping turns out to be particularly efficient for identifying natural genetic variations controlling relevant traits of yeast in an enological context [8,17–24].

One of the main limitations of this approach is the requirement of intensive genotyping and phenotyping work. While the genotyping task can be easily achieved with high throughput sequencing strategies [25,26], the measurement of complex phenotypes for several hundred individuals is not yet an easy task. Recently, various methods for measuring yeast phenotypes in a high troughtput way has been reviewed and refered to *phenomics* [27]. Although very efficient and standardized, these methods are mostly used for measuring yeast fitness (growth) by monitoring optical density (OD) or measuring colony size in various laboratory conditions. However, in food-related industry such as winemaking, many traits of interest are not related to yeast growth but are expressed during the stationary phase. Indeed, relevant phenotypic differences can be measured among strains having quite similar growth parameters [28]. Moreover, individuals showing the best growth are not always those having the highest CO_2_ production rate [11,29]. Beyond the fermentation rate, yeast also produces many metabolic compounds that affect the organoleptic qualities of the fermented beverage [18,30,31]. Therefore, an accurate assessment of fermentation-related traits remains a critical step for achieving large-scale studies including GWAS and QTL mapping.

In this study, a reliable method was set up for measuring several yeast fermentation traits in standardized 10 mL-vials. This method was useful for measuring genetics *x* environmental interactions between strains and fermentation parameters. We study here the impact of grape juice and micro-oxygenation conditions that affect most of the traits investigated. We shed light on phenotypic correlations existing between fermentation kinetics and metabolic end products by analyzing 35 commercial wine strains in five grape juices. Multivariate analyses were used for characterizing the phenotypic response of this panel. Several patterns with distinct metabolic specificities were observed and some strains are found to be more robust than other to environmental changes.

## Materials and methods

### Yeast strains and culture media used

All the yeast strains used belong to the *Saccharomyces cerevisiae* species. Four strains are monosporic clones derived from industrial wine starters that have been previously described [19,21]. The strains SB, GN and F15 are derived from Zymaflore VL1, Actiflore BO213, Zymaflore F15 (Laffort, Bordeaux, France), respectively, while M2 is derived from Oenoferm M2 (Lallemand, Blagnac, France). The remaining 31 strains used are commercial starters obtained from different companies. All these strains are genetically different and encompass the genetic diversity of wine yeast starters (Peltier *et al*. in prep). To avoid any conflict of interest there were encoded C1 to C31 and are available and deposited on the CRB collection of ISVV (S1 Table). Yeasts were propagated on YPD (Yeast extract 1% Peptone 1% Dextrose 2%) supplemented with agar (2%) when required. The strains were long-term stored in YPD with 50% of glycerol at -80°C.

### Grape musts and vessels used and fermentation monitoring

The five grape musts used, *i*.*e*. Merlot 2014 (M14), Merlot 2015 (M15), Cabernet Sauvignon 2014 (CS14), Sauvignon Blanc 2014 (SB14) and Sauvignon Blanc 2015 (SB15), were provided by *Vignobles Ducourt* (Ladaux, France) and stored at -20°C. Before fermentation, grape musts were sterilized by membrane filtration (cellulose acetate 0.45 μm Sartorius Stedim Biotech, Aubagne, France). Their main enological characteristics were determined by the wine analysis laboratory (SARCO, Floirac, France) and are given in Table 1. Sugar content was measured by infrared reflectance using an Infra-Analyzer 450 (Technicon, Plaisir, France), assimilable nitrogen as well as malic acid were assayed by enzymatic assay, total SO_2_ and free SO_2_ were assayed by pararosaniline titration. The initial active SO_2_ concentration was estimated using the protocol given at http://www.vignevin-sudouest.com/services-professionnels/formulaires-calcul/so2-actif.php. Input parameters used: pH and free SO_2_ concentration of the grape must, fermentation temperature (24°C), and 0.1% of alcohol by volume to simulate the beginning of the fermentation.

**Table 1:** Grape musts composition.

Grape must	Code	Sugar content (g.L^-1^)	Assimilable Nitrogen (mg N.L^-1^)	Malic acid (g.L^-1^)	pH	total SO_2_ (mg.L^-1^)	free SO_2_ (mg.L^-1^)	active SO_2_ (mg.L^-1^)
Sauvignon Blanc 2014	SB14	194	157	5.6	3.19	34	7	0.32
Sauvignon Blanc 2015	SB15	203	158	2.9	3.25	67	23	0.91
Merlot 2014	M14	207	111	2.1	3.58	37	29	0.54
Merlot 2015	M15	219	99	1.9	3.53	46	33	0.68
Cabernet Sauvignon 2015	CS15	220	132	2.4	3.57	35	25	0.47

To carry out the fermentations, 10 mL screwed vials (Fisher Scientific, Hampton, New Hampshire, USA ref: 11981523) were used to ferment 3 mL or 5 mL of grape must. The Screwed Vials (here after named SV) were tightly closed with 18 mm screw cap-magnetic-3mm HT silicone/PTFE (Fisher Scientific, Hampton, New Hampshire, USA). Hypodermic needles (G26–0.45 x 13 mm, Terumo, Shibuya, Tokyo, Japan) were inserted into the septum for CO_2_ release.

Fermentations were initiated by inoculating 2.10^6^ viable cell.mL^-1^ of 24h-liquid culture (YPD) carried out in 1 mL deepwell microplates (Fisher Scientific, Hampton, New Hampshire, USA). The concentration of viable cells was estimated by flow cytometry using a Cell Lab Quanta apparatus (Beckman Coulter, Brea, California, USA) according to the method described by Zimmer *et al*. [8].

The fermentation temperature was maintained at 24°C by an incubator (Binder GmbH, Tuttlingen, Germany). When specified, the SV were shaken at 175 rpm during the overall fermentation using an orbital shaker (SSL1, Stuart, Vernon Hills, Illinois, USA). In order to compare this new vessel type with already published conditions, 125 mL-glass bioreactors (GB) were also used according to the specification described by da Silva *et al*. [32].

The fermentation kinetics was estimated by monitoring manually (2–3 times per day) the weight loss caused by CO_2_ release using a precision balance (AB104, Mettler Toledo, Greifensee, Switzerland). Theoretical maximum CO_2_ release (*tCO*_*2*_*max*) was calculated according to the formula: 0.482*[Sugar] [32], where [Sugar] is the sugar concentration (g.L^-1^) of the must. The amount of CO_2_ released according to time was modeled by local polynomial regression fitting with the R-loess function setting the span parameter to 0.45. Six kinetic parameters were extracted from the model:

- *lp* (h): lag phase time observed before to release the first 2 g.L^-1^ of CO_2_;
- *t35*, *t50* and *t80* (h): time to release 35, 50 and 80% of the tCO_2_max after subtracting *lp*;
- *V50_80* (g.L^-1^.h^-1^): average hexose consumption rate between 50% and 80% of *tCO2max*;
- *CO*_*2*_*max*: maximal amount of CO_2_ released (g.L^-1^).

### Enzymatic assays

At the end of the alcoholic fermentation, a sample volume of 800 μL was manually transferred in Micronics tubes (Novazine, Lyon, France, ref: MP32033L) and stored at -20°C. The concentrations of the following organic metabolites were measured: acetic acid, glycerol, malic acid, pyruvate, acetaldehyde and total SO_2_ using the respective enzymatic kits: K-ACETGK, K-GCROLGK, K-LMAL-116A, K-PYRUV, K-ACHYD, K-TSULPH (Megazyme, Bray, Ireland) following the instructions of the manufacturer. Dilution level and volume of sample used are described in S2 Table. Glucose and fructose were assayed by using the enzymatic method described by Stitt et al. [33], however in the presented data, all the fermentations were completed containing less than 1.5 g.L^-1^ of residual sugars. All the enzymatic assays were performed by a robotic platform using the Bordeaux metabolomics facilities (http://metabolome.cgfb.u-bordeaux.fr/).

### Apolar esters analysis

Samples were analyzed after thawing. Concentration of 32 esters (ethyl fatty acid esters, acetates of higher alcohol, ethyl branched acid esters, isoamyl esters of fatty acid, methyl fatty acid esters, cinnamates and minor esters) (S3 Table). Concentration was determined using a head space solid phase microextraction (HS-SPME) followed by gas chromatography–mass spectrometry (GC–MS) as described by Antalick *et al*. [34].

### Dissolved oxygen measurement

To control the initial oxygen concentration, oxygen was removed by bubbling nitrogen inside SV for 20 min. Non-intrusive measurement of the concentration of dissolved oxygen in the grape juice was done by using NomaSense O2 P300 sensor (Nomacorc, Narbonnes, France) bonded on the inner surface of the SV.

### Statistical analyses

All the statistical and graphical analyses were carried out using R software [35]. The variation of each trait was estimated by the analysis of variance (ANOVA) using the *aovp* function of the *lmPerm* package in which significance of the results was evaluated by permutation tests instead of normal theory tests. Tukey’s honest significant difference test was used on *aovp* results to determine which group of means differ significantly using the *HSD*.*test* function (*agricolae* package) [36].

The LM1 model estimated the effect of strain, of grape must of micro-oxygenation of the strain-by-must interaction and of the strain-by-micro-oxygenation interaction on fermentation traits according to the following formula:

$$y_{ijk}=m+S_{i}+{GM}_{j}+{{MOX}_{k}+(S*GM)}_{ij}+{\left(S*MOX\right)}_{jk}+\epsilon_{ijk}$$

where *y*_*ijk*_ was the value of the trait for strain *i* (*i* = 1, …, 4) in grape must *j* (*j* = 1, 2) and with micro-oxygenation level *k* (*k* = 1, 2), *m* was the overall mean, *S*_*i*_ was the strain effect, *GM*_*j*_ the grape must effect, *MOX*_*k*_ the micro-oxygenation effect, (*S* * *GM*)_*ij*_ was the interaction effect between strain and grape must, (*S* * *MOX*)_*jk*_ was the interaction effect between strain and micro-oxygenation level and *ϵ*_*ijk*_ the residual error.

Correlations between traits were computed with the Spearman method using the *cor* function and the significance of the results was assessed by the *cor*.*test* function at 0.95 of confidence level. Results were displayed with the *corrplot* function (*corrplot* package).

Principal Component Analysis (PCA) was calculated using the *ade4* package and heatmaps were generated with the *heatmap*.*2* function. When necessary non-parametric comparison of samples were carried out using the Wilcoxon-Mann-Withney test (α = 0.05).

## Results

### Optimization of the fermentation protocol in screw capped vials

The first aim of this study was to develop a fermentation method for measuring in a reliable manner numerous strains in a small volume. We used 10 mL-screwed vials (SV) filled with 3 or 5 mL of grape must. Their small and standard size can be conveniently exploited to run in parallel more than 300 fermentations at the same time in a small space (S1 Fig). In preliminary experiments (not shown), we observed that the volume of grape juice used influences the success of the fermentation. To evaluate this effect on enological parameters, the fermentation behavior of four yeast strains (M2, F15, SB, GN) was evaluated in the SB14 grape must in six replicates. Three conditions were tested: 3 mL with shaking (Sk.3_SV), 5 mL with shaking (Sk.5_SV) and 5 mL without shaking (noSk.5_SV). In order to validate the SV, the same juice was also fermented in 125 mL glass-bioreactors (Sk.125_GB) that had been previously used for measuring the fermentation behavior of numerous *Saccharomyces* strains and hybrids [32]. For all assays, fermentations were completed (no residual sugars detected); the overall results are given in the S4 Table for the 12 parameters measured for each strain in the four assays.

**Fig 1:**
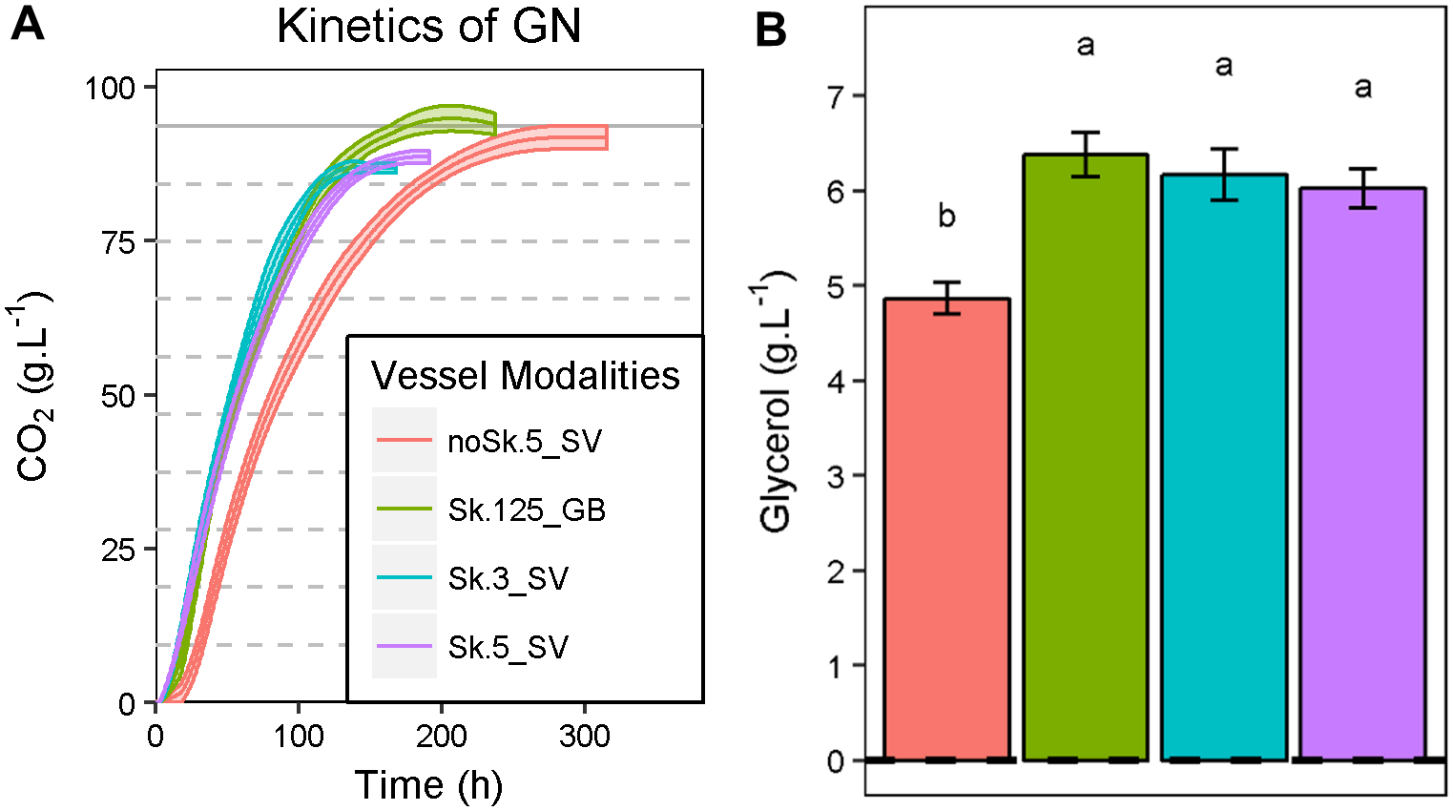
Impact of agitation on fermentation kinetics and metabolic compounds according to the fermented volume. Panel A. CO_2_ production kinetics of the GN strain fermenting SB14 grape must in four vessel modalities (Sk.3_SV, Sk.5_SV, noSk.5_SV, Sk.125_GB). The lines are the average CO_2_ produced for six replicates; the shaded areas represent the standard error. Panel B. Glycerol production of GN strain according the vessel modalities. The values shown are the means of six replicates and the error bars represent standard error.

Strikingly, the shaking conditions impacted the fermentation kinetics for all the strains. This is illustrated for example with the CO_2_ kinetics of the GN strain (Fig 1, panel A). The CO_2_ production rate was dramatically increased by shaking since the *t50* and *t80* significantly decreased (Wilcoxon test α = 0.01). In contrast, the fermentation volume (3, 5 and 125 mL) did not affect the fermentation kinetics in shaken conditions, suggesting that scaling down in SV did not influence the fermentation behavior of yeast cell. The metabolic end-products were also affected by the shaking conditions, as shown in Fig 1, panel B for glycerol. As observed for kinetic parameters, the fermentation volume had a minor impact on the primary metabolites composition (such as glycerol) whereas shaking appeared as the main source of phenotypic variation. This result, observed for all strains, could be due to the higher oxidative conditions met in shaken cultures.

**Fig 2:**
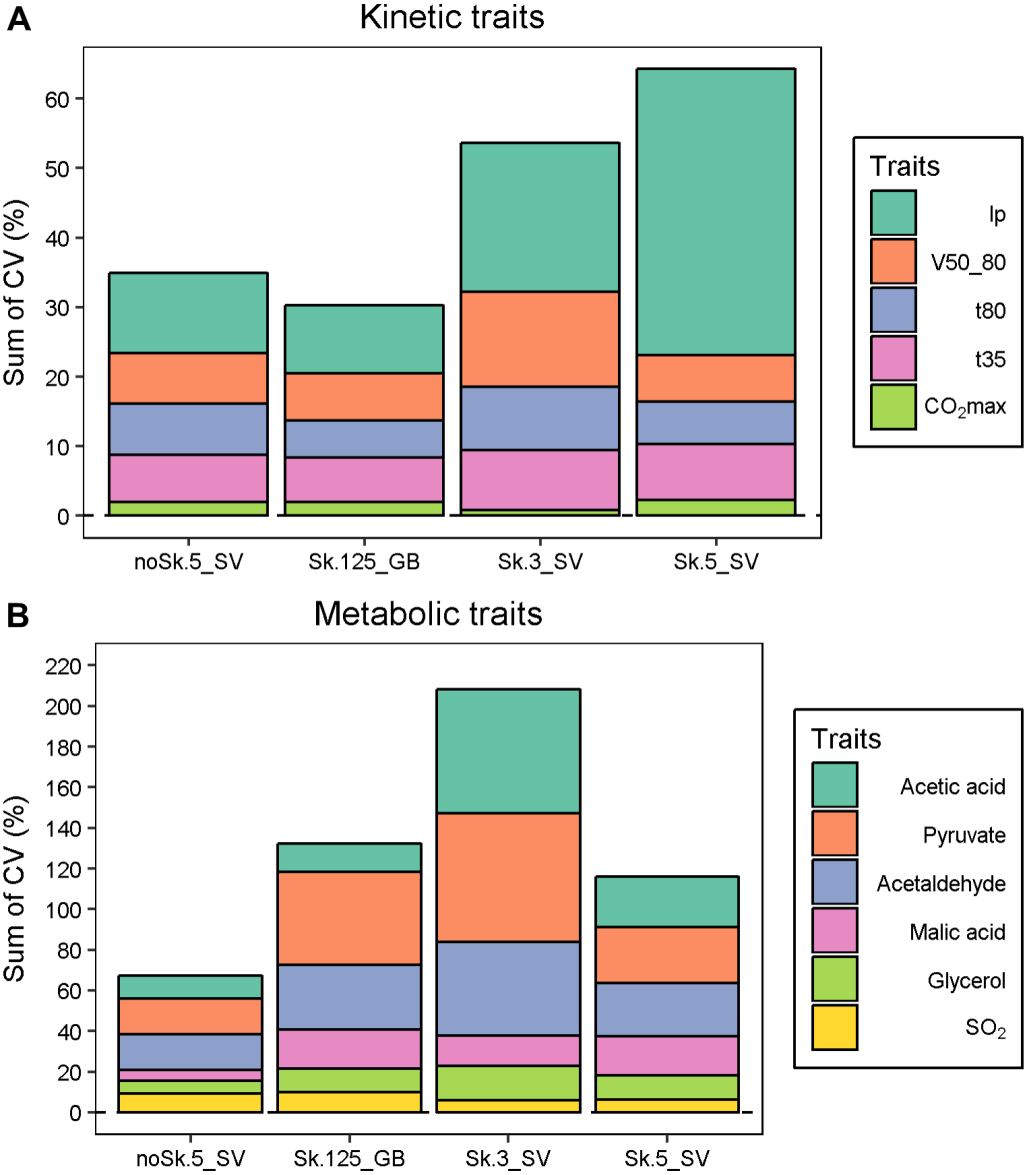
Trait measurement reliability for both kinetics and metabolite concentrations according to vessel modalities. The average CV for each trait was calculated from the CV values obtained for each strain (M2, F15, SB, GN) with six replicates. Panel A. The bar chart presents the cumulated CV for each kinetic parameter, the stacking is ordered from the least variable (*CO*_*2*_*max*) to the most variable (*lp*) trait. Panel B. The bar chart presents the cumulated CV for each metabolic end-product, the stacking is ordered from the least variable (*SO*_*2*_) to the most variable (*Acetic acid*).

To compare the reliability of the measure, the coefficient of variation (CV %) was computed for each strain and the average CV was shown in S5 Table. Each fermentation, including the Sk.125_GB, was repeated six times. The assessment of fermentation kinetic traits is very reliable and confirms the efficiency alcoholic fermentation monitoring by weight loss measurement [37], even in very small volumes (Fig 2, panel A). For some metabolic traits, high CVs (>25%) were measured showing that some conditions are not reliable enough. This is the case for acetaldehyde, pyruvate or acetic acid for which the CVs are particularly high in shaken conditions. The Sk.3_SV trial was the less reliable and the cumulated CV for metabolic compounds is much higher than for the other three conditions (Fig 2, panel B). In this condition, the kinetics parameters are also less reproducible (CV>10%). Moreover, for this condition, the concentrations of metabolites such as acetaldehyde and acetic acid are much higher than values found in enological practice (S4 Table). In contrast, noSk.5_SV offers the most reliable condition for both metabolic compounds and kinetic parameters. Except for the lag phase, the Sk.5_SV condition had an intermediate reliability level, similar to the 125 mL glass-bioreactors used here as a control.

**Fig 3:**
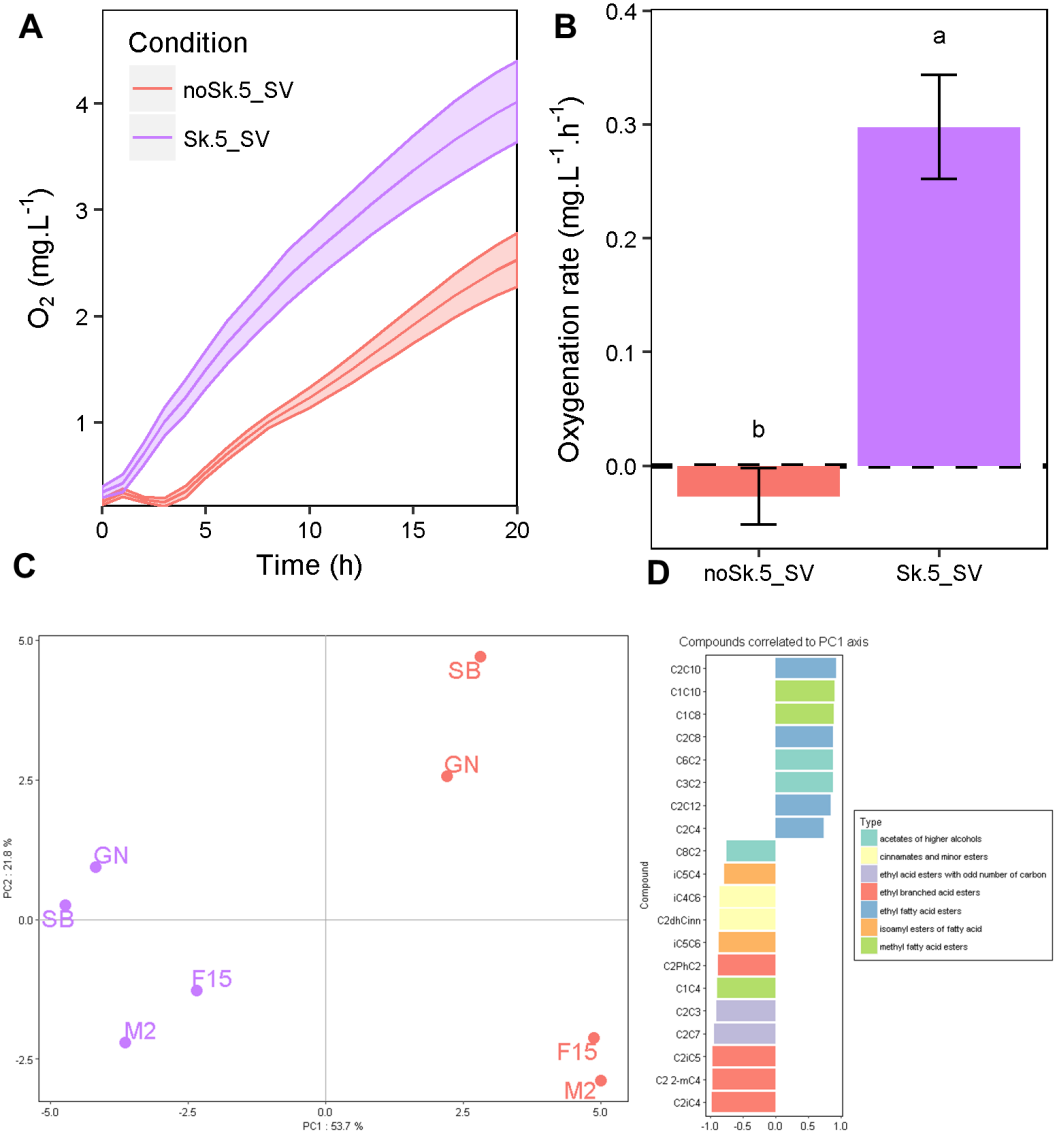
Measure and effect of micro-oxygenation in 5 mL SV. Panel A. Kinetics of dissolved oxygen concentration in SB14 grape must. The kinetic curves represent the mean of six replicates and the shadows around the lines illustrated the standard errors. Panel B. Concentration of the dissolved oxygen in SB14 after 4 h. The data shown are the means of six replicates and the error bars represent the standard deviations. Different letters indicate significant differences between groups (Tukey’s honest significant difference test, significance level, α = 0.05). Panel C. PCA performed for the 32 esters measured. Each point represents one of the four the strains in noSk.5_SV or in Sk.5_SV. Panel D. Correlation of the variables to the PCA1 axis. The variables that were significantly correlated to the first axis of the PCA were shown (α = 0.05), the bar plot indicated the Pearson’s correlation coefficient.

A second experiment was performed in 5mL-SV that enable reproducible conditions for most of the traits investigated. The 5ml-SV was preferred to 3ml-SV modality because of the outlier values and CV observed for some metabolic compounds. The micro-oxygenation effect was estimated by comparing modalities with or without shaking during the fermentation. The O_2_ concentration was monitored during 20 hours in non-inoculated SB14 grape juice degassed by nitrogen bubbling. During this period, corresponding to the fermentation lag phase, oxygen can be efficiently transferred since CO_2_ stripping is not active. Although this measurement did not correspond to real conditions since no yeast cells were present, the effect of agitation on the oxygen transfer could be estimated. Indeed, when yeast cells are present, all the dissolved oxygen is consumed in less than 20 hours due to the strong reductive conditions generated by yeast biomass (data not shown). In the shaken condition, the grape juice was immediately enriched with dissolved oxygen that reached a concentration of 3.7 mg.L^-1^ after 20 h (Fig 3, panel A). In contrast, without shaking, there was only 2.4 mg.L^-1^ of dissolved oxygen after 20 h. A maximum difference in oxygenation rate was found after three hours of incubation (Fig 3, panel B). Although the total amount of oxygen transferred during the overall fermentation cannot be measured, these data suggest that agitation in 5mL-SV significantly impacts the micro-oxygenation level. These small, but significant differences could explain the kinetic and metabolic differences described in Fig 1.

In order to have a broader idea of the impact of micro-oxygenation on secondary metabolism, we next measured the production of volatile compounds. At the end of the alcoholic fermentation, the headspace volume of SV was analyzed using a targeted GC-MS analysis. 32 esters were quantified for the fours strains in shaken or not conditions (S6 Table). A Principal Component Analysis (PCA) (75.5% of total variance for axes 1 and 2) was carried out for exploring this multivariate dataset (Fig 3, panel C). The first component clearly discriminates shaken from non-shaken conditions while the second axis mainly discriminates strains. Indeed, the production of esters was greatly impacted by shaking. Up to 27 of the 32 esters were significantly affected (ANOVA, pval<0.05), 14 with a decreased and 13 with an increased production in the shaken condition (S6 Table). The compounds, for which shaking decreased their production, were mainly acetates of higher alcohols, methyl and ethyl fatty acid esters while those for which the production was increased were mainly ethyl branched acid esters, ethyl acid esters with odd carbon numbers, cinnamates and minor esters. The proportion of PhC2C2 to C2PhC2 was six-fold decreased in shaken condition (S2 Fig). This could be caused by a higher oxygenation of the media.

### Assessment of genetics x environmental effects

**Fig 4:**
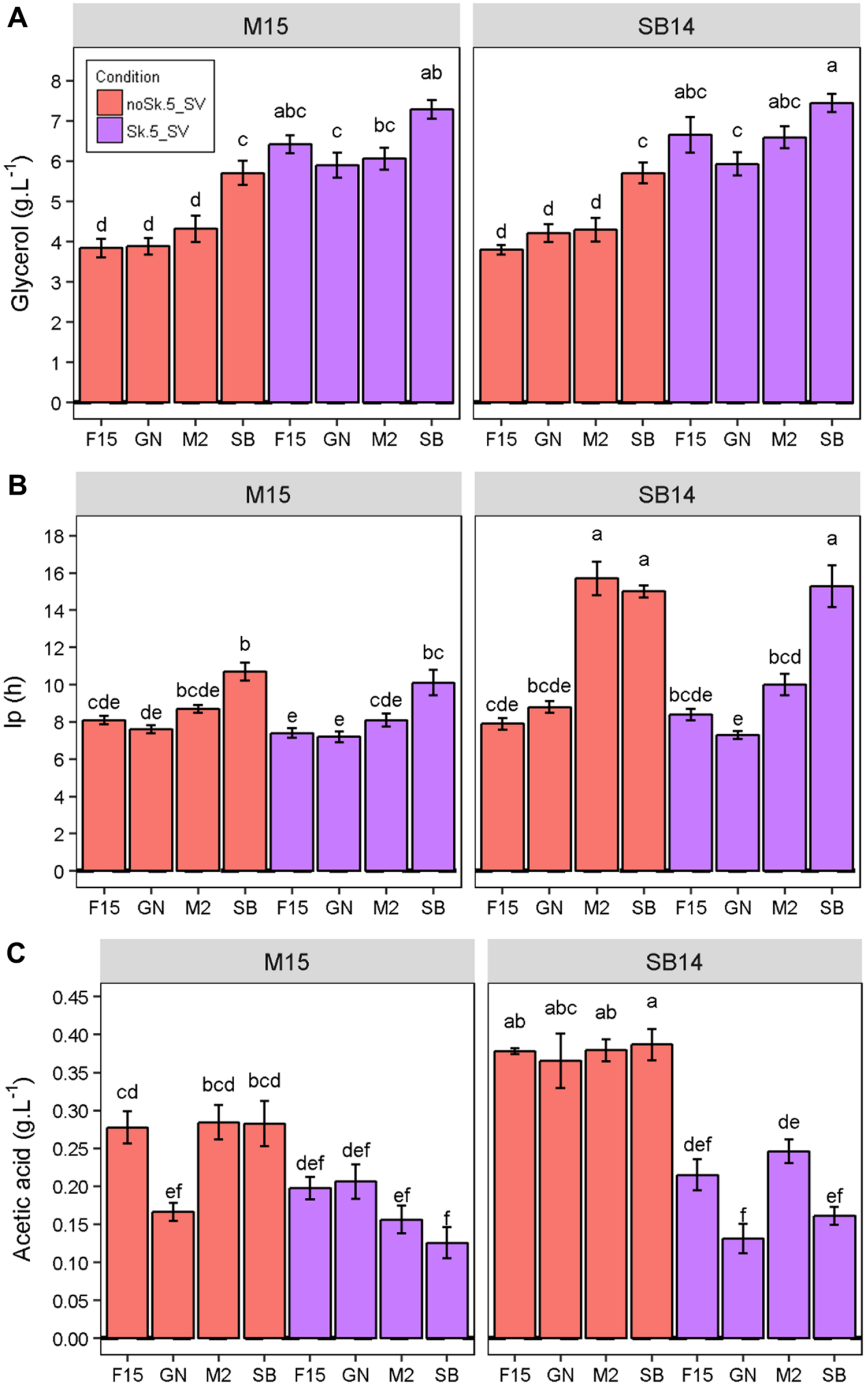
Effect of micro-oxygenation level and grape must on technological properties of wine yeast strains. The data shown are the mean of ten replicates, the error bars representing the standard error. Different letters indicate significant differences between groups (Tukey’s honest significant difference test, significance level, α = 0.05). Panel A. *Glycerol* (g.L^-1^) according to strain and fermentation conditions. Panel B. *lp* (h) according to strain and fermentation conditions. Panel C. *Acetic acid* (g.L^-1^) according to strain and fermentation conditions.

In order to demonstrate the efficiency of our SV fermentation setup, we explored phenotypic response of strains to relevant parameters for enology. On the basis of the results shown in Figs 2 and 3, shaken fermentations could be considered as micro-oxygenated modalities transferring moderate amounts (2–4 mg.L^-1^ per day) of oxygen in a reproducible way. The possibility to control oxygenation in small volumes is an opportunity to study the reaction of yeast strains against this technological parameter which has a significant impact on winemaking [38–40]. Assuming this statement, a second experiment was carried out in 5-SV, by fermenting the two grape juices SB14 and M15 with four strains (M2, GN, F15 and SB) and with or without shaking. This set of 160 fermentations (S7 Table) ran at the same time allowed to estimate the effects of three main factors: (i) strain, (ii) micro-oxygenation, and (iii) grape must. The proposed model for the analysis of variance also estimated the primary interaction within strain and grape must or micro-oxygenation (model LM1 described in Material and methods). Thanks to the small volume used, ten biological replicates were carried out for each strain and condition, thus increasing the statistical power of the analysis. For most of the traits, the phenotypic variance was first explained by the grape juice nature, then by the yeast strain used (Table 2). The effect of micro-oxygenation mainly influenced kinetic parameters (*t50*, *t80*) and metabolic end-product such as *SO*_*2*_ and *Glycerol*. For this last trait, the micro-oxygenation increased the production by 15% (Fig 4, panel A) for all the strain, as previously reported by others [41–43]. Few strain *x* environment interactions were detected and accounted only for a small part of the total variance explained (less than 10%). The most striking interaction effects were found for the lag phase duration (*lp*). Indeed, for this trait, the yeast strain phenotype is differentially affected by both the micro-oxygenation and the grape must. The panel B of Fig 4 shows that the strains SB and M2 had a longer lag phase in the SB14 grape must than in M15 (+ 6 h). Moreover, shaking resulted in a reduced lag phase for M2 in the SB14 grape must. In contrast, F15 and GN were affected neither by the grape must nor by the agitation. In the same way, the *acetic acid* production of GN showed a complex GxE interaction (Fig 4, panel C). Globally, as previously described [44], micro-oxygenated conditions tended to reduce the production of this compound, which is undesirable in enology. Interestingly, in the M15 grape must, GN showed the lowest acetic acid production even in a non-agitated fermentation, suggesting that it is an interesting lower producer whatever the conditions. This second experiment confirms the reliability of SV for assessing wine fermentation traits in various environmental conditions and paves the way for larger phenotypic investigations.

**Table 2:** Analysis of variance for the eleven phenotypes with four strains, two musts and two micro-oxygenation conditions.

	CO_2_max	lp	t35	t50	t80	V50_80	SO_2_	Acetic acid	Malic acid	Pyruvate	Glycerol
Must	37.9 ***	15.8 ***	38.6 ***	36.8 ***	35.2 ***	21.5 ***	15.4 ***	10.7 ***	81.2 ***	4.9 **	0.3
Strain	2.3	41.5 ***	10.3 ***	16.7 ***	27.3 ***	43.8 ***	4.3 ***	3.7 *	8.1 ***	9.0 ***	17.2 ***
Micro-Oxygenation	7.4 ***	2.8 ***	37.2 ***	32.1 ***	22 ***	20.2 ***	40.3 ***	39.1 ***	0	5.2 ***	49.4 ***
Strain:Must	0.3	10 ***	2.1 ***	2.5 ***	2.7 ***	0.5	2.8 **	0.4	0.2	6.8 ***	0.1
Strain:Micro-Oxygenation	0.6	3.7 ***	0.7 *	0.3	1.6 ***	0.1	1.8	2.6 *	1.1 ***	4.0	1.8 *
Residuals	51.4	26.2	11.1	11.6	11.3	13.9	35.5	43.5	9.4	70	31.3

Percentage of variance explained by the LM1 model. Signifiance codes: pval < 0.001 = ***, pval < 0.01 = **, pval < 0.05 = *, pval < 0.1 =.

### Evaluation of technological properties of 35 wine yeast strains in five grape juices

The SV fermentation setup coupled with robotic assisted enzymatic assays offers the opportunity to measure in parallel the fermentation behavior of numerous strains in various conditions. As a matter of proof, we evaluated in a unique experiment the fermentation properties (kinetics and end by-products) of 35 strains in five grape juices and two repetitions (350 SV) without shaking. No shaken conditions were preferred in this experiment because they emphasize the phenotypic differences between strains due to the hypoxic conditions generated (Fig 1, S4 Table). In this experiment, we used three red grape musts (M14, M15 and CS14) and two white grape musts (SB14 and SB15) from the Bordeaux area. As all the fermentations were completed (less than 1.5 g.L^-1^ of residual sugars), the final concentrations of glucose and fructose were very low and thus removed from the data (not shown). Acetaldehyde concentrations were also removed, as they were very low in red wines and thus impacted data normality (not shown). The measurement of the 11 quantitative variables for 175 modalities is given in S8 Table.

**Fig 5:**
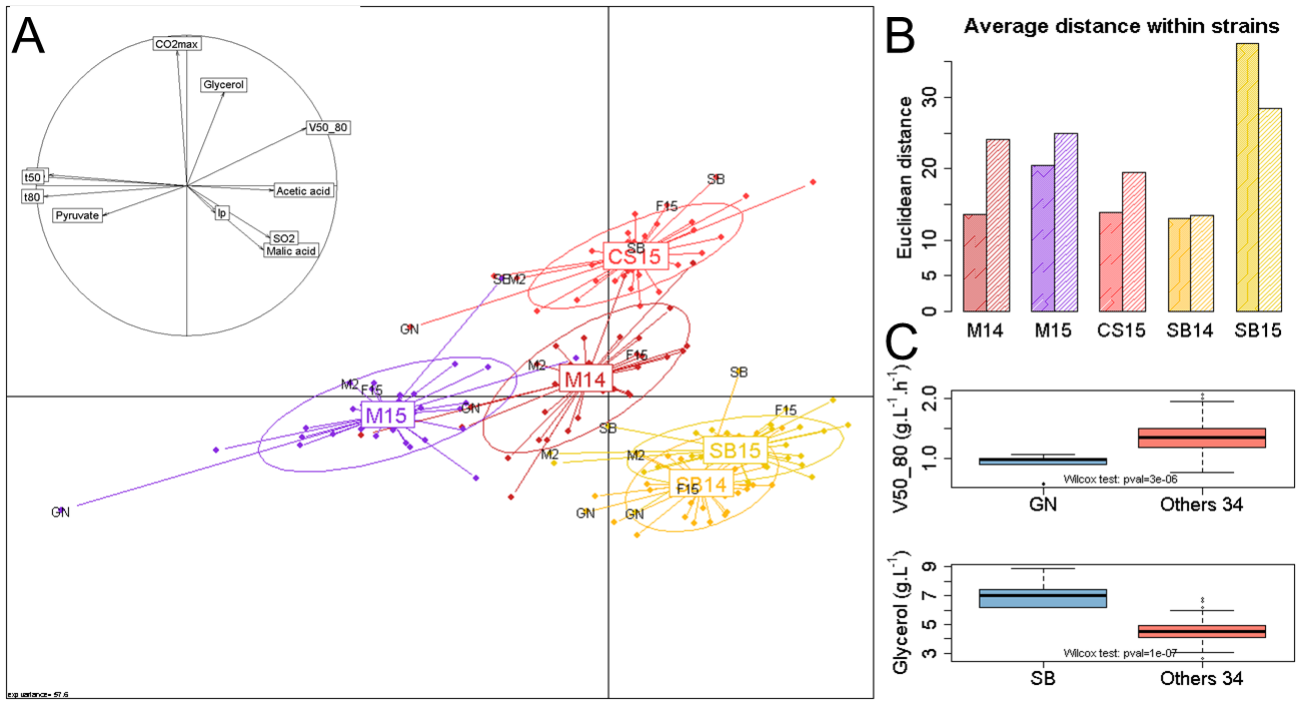
PCA of winemaking properties of 35 strains in five grape juices. Panel A. The first two axes of the PCA performed from the average of two replicates for 11 phenotypes measured in the five grape juices and 35 strains. Axes 1 and 2 explain 41.8% and 15.8% of total variation, respectively. Each point represents the fermentation of one strain and is colored according to the grape juice used. Points are connected to their group gravity centers that are labeled with the grape juice name M14, M15, SB14, SB15, CS14. Ellipses diameter corresponds to the standard deviations of the projection coordinates on the axes. The correlation circle indicates the correlation of the variables for axes 1 and 2. Panel B. Euclidian distances within all the strains for each grape must. The bar plot represents the Euclidian distances within the 35 strains according to kinetics (high density colored bar) and metabolic parameters (low density colored bar) for each grape juice. Panel C. Comparison of the trait value of GN and SB respect to the 34 others strains for V50_80 and the glycerol produced, respectively. A boxplot was generated from the ten phenotypic values measured in the five grape juices with two replicates for GN and SB, and from the 340 values of the 34 other strains. Significant differences were estimated by applying the Wilcoxon-Mann-Withney test (α = 0.05).

This large dataset offer the possibility to address several questions. First we explores the impact of the grape juice nature by performing a multivariate analysis. The first two axes of the Principal Component Analysis (PCA) carried out explained 58% of total variance. The first component (42% of total variance) clearly discriminates red and white juices and was correlated with *Malic acid*, *SO*_*2*_, *Acetic acid* concentrations and kinetic parameters (*t50*, *t80*, *V50_80*) (Fig 5, panel A). Indeed the white grape juices used were more acidic and more sulphited than red ones. The second axis (16% of total variance); mainly discriminates the CS15 must from the others by its higher production of *glycerol* and *CO*_*2*_*max*. These results are consistent with the biochemical composition of grape juices since the CS15 juice contained 20 g.L^-1^ more sugar than the other grape musts.

The PCA also illustrates the phenotypic variability of the 35 industrial strains tested. Globally, the analysis showed that some grape musts are more suitable than others for differentiating the strains. Indeed, the projected cloud of the 35 strains in SB14 is more compact than in M15. In order to evaluate this property, we computed the average Euclidian distance within all the strains for both kinetic and metabolic parameters and according to the grape must. The panel B of Fig 5 summarizes the phenotypic distance observed within each grape must and each parameter class. For example, SB15 emphasized strain discrepancy for kinetic traits and metabolic end-products. To better visualize particular strain properties, the positions of the four strains SB, GN, M2 and F15 were labeled on the projection. These strains have some phenotypic specificities; for example SB and GN are often more distant from the remaining set of commercial strains than M2 and F15. This is in particular due to the high *glycerol* production of SB and the slow fermentation rate (*V50_80*) of GN in all the conditions tested (Fig 5, panel C).

**Fig 6:**
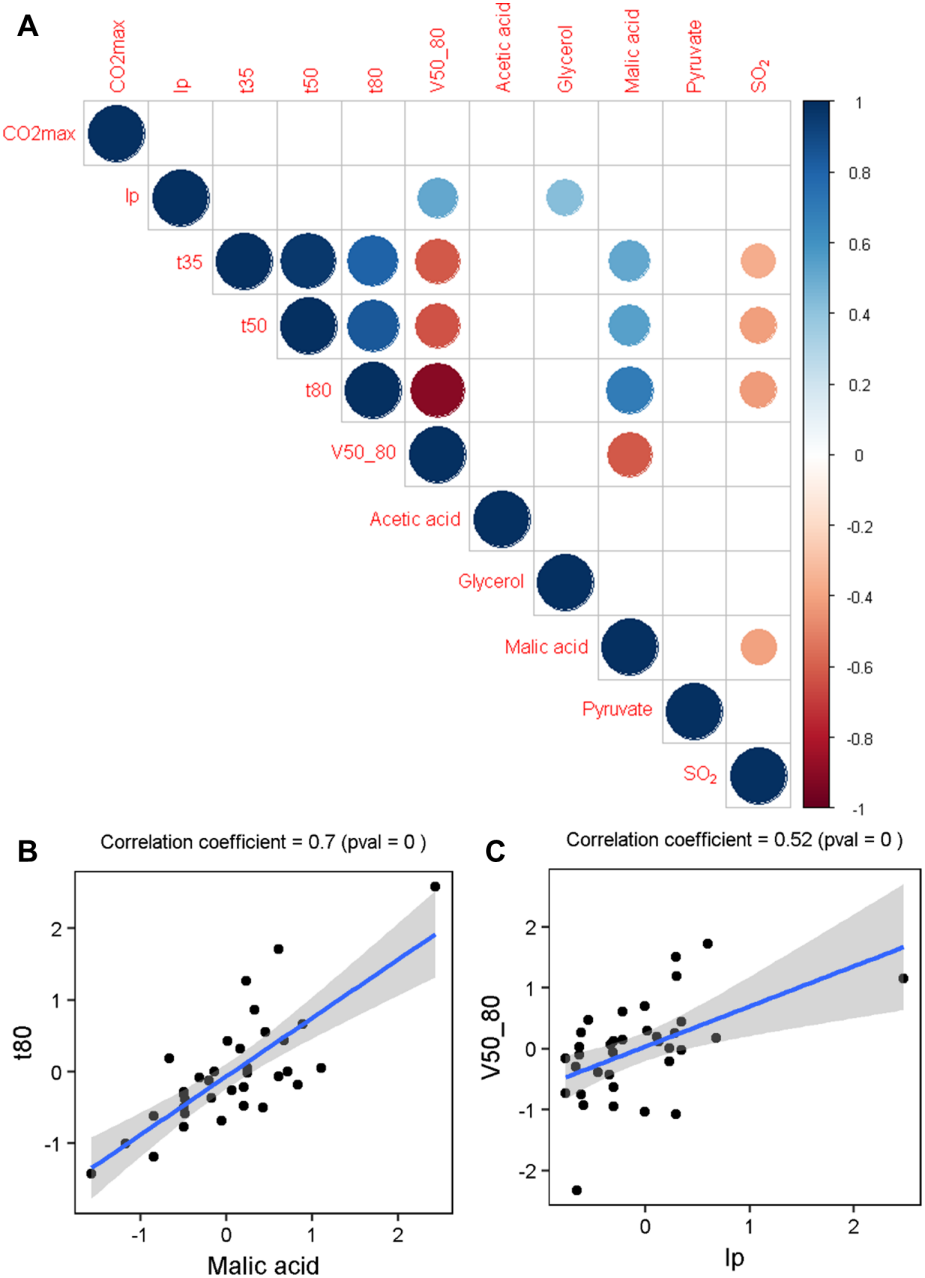
Correlation between traits. Panel A. A correlation matrix is shown. The size and the colour of the circles correspond to the correlation coefficients calculated by the Spearman method. Only significant correlations are shown (confidence = 0.95). Panel B and C. Two examples of scatter plots showing correlation of *t80* with *Malic acid* and *V50_80* with *lp*. Each dot represents the average phenotypic values of a strain across the five grape musts from the normalized dataset. The blue line represents the linear regression line and the shaded area represents the confidence interval of the regression (0.95).

In a second time we analyzed the phenotypic correlations existing between the eleven quantitative traits investigated. As shown on the PCA, the nature of the juice strongly impacted the phenotypic values. In order to overcome this effect, we normalized the response of each strain according to the grape juice (S9 Table). This normalization confers a stronger robustness to the analysis since the phenotypic properties of the strains are considered across five conditions. However, using this transformed dataset, all the strain *x* environment interactions are hidden. The relations between the eleven traits were investigated by using the average of normalized values of each strain for the five conditions. The Spearman’s correlation matrix computed was shown in the Fig 6, panel A. Obvious correlations between kinetic traits were found confirming that the fast-fermenting strains have the lowest t35 and t80 values (S3 Fig). Interestingly, we detected less trivial correlations suggesting some possible connexions between metabolic end products and kinetics parameters. For example, a correlation between *t80* and *Malic acid* was found (Fig 6, panel B). Indeed fast fermenting strains are those that consume the most part of malic acid. This link has already been reported [45] and could be explained by a greater deacidification capacity for strains that consume more malic acid, resulting in easier fermentation. Negative correlations were found between kinetic parameters (*t35*, *t50*, *t80*) and *SO*_*2*._ These negative relations could be explained by the toxic effect of SO_2_ that reduces yeast growth [46,47] and may indirectly impact the fermentation activity. Other correlations were found for *lp* with *V50_80* (Fig 6, panel C) and *glycerol* and will be discussed further.

**Fig 7:**
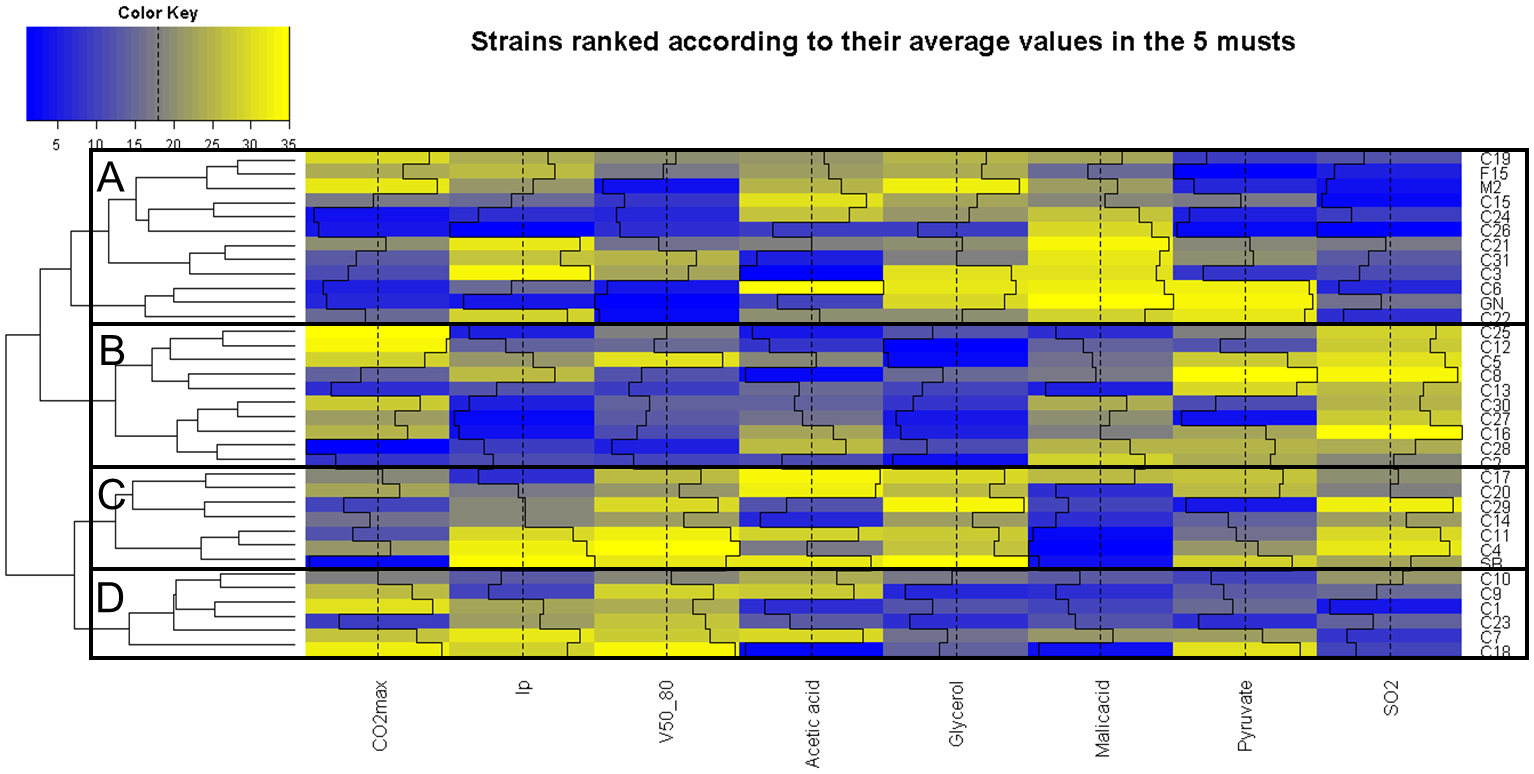
Relative ranking of 35 strains in five grape juices. Ascending order ranked of the average phenotypic values of each strain across the five grape juices. Only a subset of the representative phenotypes is represented here. A color palette shows each rank from blue (lowest ranks) to yellow (highest rank) as displayed by the color key. The rank of each cell is also displayed by a black bar plot and the vertical dashed black line represents the average rank. The dendrogram on the left represents strain ordered by hierarchical clustering.

The normalized dataset was also used for evaluating the technological properties of the 35 strains in a multi environment context. As for the phenotype-phenotype relation study, the use of normalized data confers more robustness to the analysis. The rank of each strain with respect to the others was calculated and can be visualized on a heatmap plot (Fig 7). As each column of the heatmap plot represents a rank value (1 to 35), each trait has the same weight in the clustering. Because most of the kinetic parameters are strongly correlated (Fig 6, panel A), only three of them (poorly correlated) were included in the analysis (CO_2_max, *lp* and *V50_80*). The intensive blue tones indicate lowest ranks while intensive yellow tones indicated the highest ranks for each parameter. For example, the commercial strains C11, C4 and C18 were among the fastest strains and consumed more malic acid than the others. Rapid identification of strains having outlier levels compared to a representative commercial set can be made with this figure. For example, the strains C6, C17 and C20 produced high quantities of acetic acid while the strains C5, C8 and C16 released an important quantity of SO_2_ at the end of the alcoholic fermentation.

As displayed by the dendrogram on the left of the heatmap, a hierarchical clustering ordered the strains according to their overall profiles. Four main groups could be discriminated. The group A contained slow fermenting strains, which leave high amounts of *malic acid* at the end of the fermentation and produce low *SO*_*2*_. Group B contained strains with the shortest *lp*. Moreover, most of the strains of this group had a slow fermentation rate, produced low amounts of *glycerol* and released high level of *SO*_*2*_. The strains of group C were the fastest fermenting ones, produced more *glycerol* and *SO*_*2*_ than the average. This group also consumed more *malic acid*. Finally, the strains of group D fermented rapidly but in contrast with those of group C they produced low amounts of *glycerol* and *SO*_*2*_.

**Fig 8:**
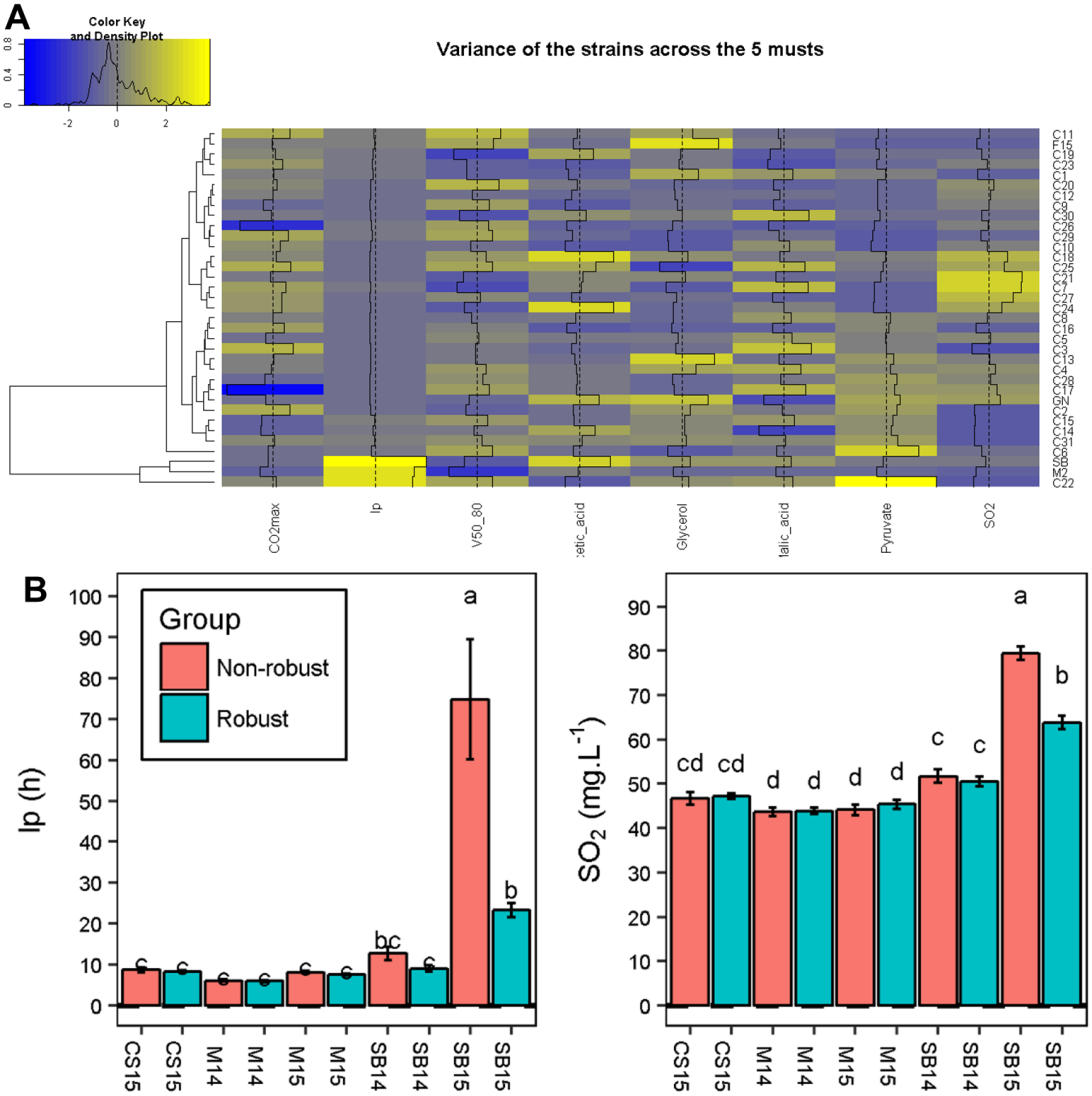
Phenotypic variance of 35 strains in five grape juices. Panel A. For each strain, the variance was computed for the five average phenotypic values in the five grape musts. Variance is scaled by column and its level is represented by a color palette from blue (lowest variance) to yellow (highest variance) as displayed by the color key. The value of each cell is also displayed by black bar plots and the vertical dashed black lines represents the average variance. Strains are ordered by hierarchical clustering that is represented by the dendrogram on the left. Panel B. Comparison of *lp* and *SO*_*2*_ between robust and non-robust strains according to grape musts. The data shown are the mean of eight strains (non-robust group) or 27 strains (robust group), the error bars represent the standard error. Different letters indicate significant differences between groups (Tukey’s honest significant difference test, significance level, α = 0.05).

Finally, we investigated the strain phenotypic variability according to the environmental conditions. This characteristic is very important in enology since industrial strains might be used in different grape musts with contrasted physicochemical properties. Therefore, the assessment of phenotypic robustness of industrial starters is crucial for optimizing their use in a wine making process. We computed the phenotypic variance of the 35 strains by using the non-normalized dataset, which allows capturing the GxE interactions. The overall results are shown on Fig 8, panel A. Strains showing a low variance value (blue tones) had similar phenotypic behavior in the five grape musts and can be considered as robust. On the contrary, high variance values (yellow tones) indicated a fluctuating phenotypic response according to the must. Some industrial strains such as C23, C10 or C12 are quite robust to environmental change. In contrast, the monosporic clones SB, GN and M2, as well as some commercial strains (C22, C7, C18) appeared to be quite sensitive to the grape must nature (yellow tones). This excess of sensitivity was investigated by splitting the 35 strains in two groups according to their phenotypic variance. The more fluctuating quartile was compared to the 75% more robust strains in the five grape juices. By this way, the conditions that generate a fluctuating response could be identified. For example, *lp* was only significantly different for the two groups in SB15 (3.2 time shorter for the robust group) (Fig 8, panel B). In this case, the identified grape must had the strongest initial SO_2_ concentration (67 mg.L^-1^), which is known to strongly affect the lag phase [8]. All the strains having a fluctuating response (C1, C11, F15, C15, M2, C22, SB, C31) are therefore not suitable for running fermentations in highly sulphited grape musts. In the same way, another group of strains (C4, C7, C17, GN, C18, C21, C24, C25, C27) produced high concentrations of *SO*_*2*_ at the end of the fermentation only in the SB15 must (Fig 8, panel B). Merlot (M14 and M15) and Sauvignon Blanc (SB14 and SB15) musts increased the variance of fluctuating strains for *pyruvate* and *acetic acid*, respectively (S4 Fig). The strains C18 and C24 produced high levels of *acetic acid* in white grape musts but they showed a moderate production in the three red grape musts suggesting that are more suitable for red wine fermentation.

## Discussion

### A new platform for measuring quantitative traits related to wine fermentation

The wide development of high throughput sequencing technologies gives the opportunity to collect large sets of genomic data that could be used for dissecting the genetic architecture of complex traits using both QTL mapping and GWAS approaches [16]. In order to implement genetic studies efficiently, this genomic data must be completed with massive sets of phenotypic data. The high throughput measurement of phenotypes is therefore a crucial point for finding out new genetic determinisms. In the last decade, the term of “phenomics” has been used to describe methods aiming at measuring phenotype at a large-scale [27]. Mostly based on the measurement of OD [48] or plate growth [49], the parallel measurement of basic growth parameters in numerous media can be performed. Although this approach is very useful for screening growth-related phenotypes, other complex traits of industrial interest, such as fermentation kinetics and end-product metabolites can neither be measured in micro-plates nor in agar plates.

In this study, we set up a standardized method for assessing alcoholic fermentation experiments at a relatively large scale (>300 samples per batch). By reducing the fermentation volume to 5 mL in standard Screwed Vials (SV), we conserved a very accurate estimation of fermentation kinetics that matches well with the methods previously used [50]. Here, the fermentation time course was followed manually by weighing each SV two times per day with a precision balance. However, robotic solutions for an automatic handling of the SV could easily be implemented thanks to the standardized format of the vials used. In order to face the large sample analysis set required, we successfully coupled our fermentation setup with a robotized enzymatic platform for measuring eight enological metabolites in 1 mL samples. The assay of other compounds of enological interest could be implemented with our system including ascorbic acid, citric acid, lactic acid or assimilable nitrogen. Unfortunately, we failed to efficiently measure ethanol, since the enzymatic kit used was not sufficiently accurate for high ethanol concentrations. Alternatively, the estimation of total CO_2_ loss was very precise (average CV<3%) and perfectly matched with the production of ethanol during the alcoholic fermentation [37]. In this study, we also demonstrated that many volatile compounds produced by yeast metabolism could be readily analyzed by GC-MS after an automated solid-phase micro-extraction [34]. Coupling analytical facilities and developing robotic handling of SV will be the next steps for developing large screening programs.

### Assessment of some GxE interactions relevant in enology

Although the SV volumes are far away from those of vats used during industrial wine production, our setup came close to meeting the enological conditions. The effects of some parameters that are relevant for enology (grape must, strain, micro-oxygenation level) could be tested. First of all, we used natural grape musts rather than synthetic media that might be less pertinent for assessing quantitative traits due to their incomplete composition [21,51]. As previously demonstrated, frozen grape juices conserved their fermentation properties and can be kept for long periods [32]. Moreover, in this work, we only tested a panel of commercial starters that are used in various geographic areas for the production of red, white, *rosé* and sparkling wines. This contrasts with previous studies that also included *S*. *cerevisiae* strains from other origins [52,53]. By using only commercial starters, we captured here a phenotypic variability having an industrial relevance and reflecting those proposed to the winemaker. Finally, the shaking of SV was able to mimic micro-oxygenation in a reproducible manner. The amount of oxygen transferred during the 20 first hours (2–4 mg.L^-1^ of O_2_) is close to that can be applied during red winemaking [40]. Although the micro-oxygenation is provided by several pumping-over operations in the cellar, we were able to reproduce this effect in our small design vessels with similar scale values. This was confirmed by observing effects that are similar to those already known in enological practices. Indeed, a higher level of micro-oxygenation accelerates the fermentation rate [40,54,55], decreases the production of acetic acid [44,54], and increases the production of glycerol [41,42,54,56].

Although the micro-oxygenation enhances the basic technological properties of yeast, it also impacts drastically the production of volatile compounds. By using a GC-MS approach we demonstrated that shaken conditions do not impact all the volatile molecules in the same way suggesting that the oxygen transfer could influences the production of aromatic compounds and in particular esters. The change in proportion of 2-phenylethyl acetate (2-PhC2C2) to ethyl-phenylacetate (C2PhC2) could be a signature of micro-oxygenation, as the proportion of the most oxidized ester (ethyl-phenylacetate) is greater with agitation (S2 Fig). Moreover unusual esters such as ethyl dihydrocinnamate (C2dhCinn) or methyl butyrate (C1C4) are much more present in shaken fermentation (Fig 3, panel C and S6 Table). This results indicated that shaken conditions could lead to an over oxidation at least in white grape juice matrixes. This result justify our choice to phenotype the 35 strains in unshaken conditions.

Unravelling the impact of oxygen on esters production during the alcoholic fermentation is not trivial. First, it is difficult to conclude here if the oxygen has a direct effect on ester metabolism. Indeed oxygen could simply enhances yeast growth and/or accelerates the fermentation rate which in turn may have an effect of ester production. Second, shaking might increase the liquid to gas transfer enhancing the volatile compounds stripping. According to the quantity and the addition moment, the oxygen effect may indeed be drastically different. The oxygen supplementation of grape must in winemaking conditions resulted in an increase of the concentration of higher alcohol acetates and branched chain ethyl esters, and in a decrease of fatty acid ethyl esters [54,57]. Aside higher alcohol acetates that were two times higher in non-shaken conditions, our findings are broadly in agreement with previous data measured in a cellar [54,57]. The similar response between 5mL-SV and vats of several liters is very encouraging and demonstrates that our setup could be relevant for assessing the aromatic production of a large set of strains/conditions. Moreover, the relative higher production of acetate of higher alcohols in non-shaken conditions could be explained by the fact that the moment of oxygen addition and metabolizing is drastically different between the yeast growth and stationary phase [38,58]. For example, in a brewing context, when oxygen is added during the fermentation, a decreased production of higher alcohol acetates can be observed [58,59], thus supporting our observations (S6 Table). Conversely, oxygen addition has been reported to increase the concentration of ethyl esters and to reduce the concentration of acetate esters and higher alcohols [60]. These seemingly contradictory results can be also due to strain-by-oxygenation interactions. Indeed 16 of the 32 compounds assayed showed *strain x environment* interactions. This method could be therefore useful in the future to better investigate the physiological and enological consequences of micro-oxygenation for up to very large panels of yeast strains.

Thanks to this setup, we gained insight on other GxE interactions between wine strains and environmental conditions. For example, GN maintained a constant level of *acetic acid* in M15, regardless of the level of micro-oxygenation. This particular feature, which is a relevant trait in enology, suggests that acetic acid metabolism is poorly impacted by hypoxia in this strain. Another interaction was observed for the strain M2, for which the long lag phase observed in sulphited grape must (SB14) is reduced by the micro-oxygenation. Those preliminary observations open perspectives for studying the phenotypic response of yeast strains to micro-oxygenation at a large scale.

### Survey of the fermentation properties of 35 enological strains in five grape musts

As a matter of proof, we measured the technological properties of 35 strains including 31 industrial starters. After three weeks of fermentation, we measured in the same batch 11 traits in five different grape juices (350 fermentations), supporting the efficiency of the method for high throughput phenotyping. We have observed an important grape must effect on the phenotypes (Table 2). This effect was generated by the basic physicochemical characteristics of the grape musts (concentration of sugar, malic acid, and SO_2_ *etc*.). In order to go beyond this effect, the response of each strain was normalized according to the grape juice eliminating the media effect but also possible GxE interactions. The correlation analysis of traits measured in five conditions reinforces the robustness of these links and ensures the generalization of the conclusions that can be drawn. Interestingly two relevant correlations were found. A strong relation was identified between *malic acid* and kinetic parameters (*T35*, *T50* and *T80*). Thus, fast fermenting strains were also those consuming more malic acid. This had already been reported in an isogenic context for the ML01 strain, which has been genetically modified to carry out the malolactic fermentation [45]. ML01 has a higher fermentation rate than the parental strain due to the deacidification of the media caused by the malic acid consumption. However, this effect was reported only in low pH conditions. Because in our study the pH of grape must range from 3.19 to 3.58, other mechanisms might be involved. For example it is known that malic acid plays an important role in carbon metabolism. During fermentation, its decarboxylation provides pyruvate which could play an anaplerotic effect on biomass and/or on ethanol synthesis [61,62]. A second positive correlation was found between the duration of the lag phase (*lp*) and the glycerol production, suggesting that strains with long lag phase produce more glycerol. At the beginning of fermentation, the glycerol production is critical for restoring the redox balance by regenerating the NAD^+^ consumed by the glycolysis [63]. Indeed, at that stage, the regeneration of NAD^+^ by alcohol dehydrogenase is inhibited by the formation of acetaldehyde-SO_2_ complexes. Thus, strains unable to initiate rapidly the alcoholic fermentation have to cope with NAD^+^ depletion and may have be selected for their high glycerol production level.

The dataset generated was also useful for evaluating the technological performance of the strains. This comparison revealed groups of strains with distinct phenotypic profiles. For example, all the strains of the groups C and D (Fig 7) have the highest fermentation rate but are mainly discriminated by their *glycerol* and *SO*_*2*_ production levels. This might suggests that commercial strains well adapted to winemaking conditions have undergone different adaptive routes that have modelled their central metabolism. The phenotypic robustness of strains fermenting in various grape juices has been also evaluated. Although this characteristic has been poorly investigated in the past, robustness is a critical factor to take into account in yeast selection. Surprisingly, only few strains are robust to environmental changes and are able to ensure stable phenotypes in a wide range of grape musts. Indeed most of the strains showed fluctuating phenotypes according to the grape must used. The use of grape musts with extreme characteristics (SO_2_ or sugar concentrations) highlighted the weakness of the less robust ones leading to the identification of the type of grape must for which they are the most suited. The setup developed in the present study could help to identify the physicochemical factors (amino acids, vitamins, cofactors or polyphenols) that could be a source of inappropriate phenotypic responses. The identification of enological factors that affect the performance of strains is of great interest. It has already been shown for example that the effect of temperature during fermentation was dependent on the strain used [28]. The fermentation system implemented here is well adapted to push forward the identification of new factors of this type.

## Supporting information

S1 Fig SV setup. On the left, a SV filled with 5 mL of grape juice (SB14) and with a hypodermic needle to allow the CO_2_ release. On the right 70 vials on a rack illustrating the possibility of managing hundreds of fermentations in parallel.
(TIF)

S2 Fig Oxygen impact on ester production. Panel A. The data shown are the mean proportion of PhC2C2 to C2PhC2 of the four strains in two replicates, the error bars represent the standard error. Different letters indicate significant differences between groups (Tukey’s honest significant difference test, significance level, α = 0.05). Panel B. The data shown are mean of two replicates, the error bars represent the standard error. Different letters indicate significant differences between groups (Tukey’s honest significant difference test, significance level, α = 0.05). Table represents ANOVA results (pval, and % of variance explained).
(TIF)

S3 Fig Correlations between traits. Scatter plots of correlated traits. Each dot represent the average phenotypic values of a strain across the five grape must from the normalized dataset. The blue line represents the linear regression line and the shaded area represents the confidence interval of the regression (0.95).
(TIF)

S4 Fig Comparison of the phenotypic values between robust and non-robust strains according to grape musts. The data shown are the mean of eight strains (non-robust group) or 27 strains (robust group), the error bars represent the standard error. Different letters indicate significant differences between groups (Tukey’s honest significant difference test, significance level, α = 0.05).
(TIF)

S1 Table Yeast strains used. (XLSX)

S2 Table Dilution and volume of sample used for robotic enzymatic assay. (XLSX)

S3 Table List of the 32 esters analyzed. (XLSX)

S4 Table SB14 dataset. Data presented are the mean of six fermentation replicates of SB 14 grape must. The residual sugars (glucose + fructose) at the end of the fermentation was not shown and was always lower than 1.5 g.L^-1^. Statistical differences within strains and modalities was assayed by Tukey’s honest significant difference test, significance level, α = 0.05, the different groups were shown by a letter code: groups sharing the same letter are non-significantly different.
(XLSX)

S5 Table Average coefficient of variation for the different traits. The data presented are the average coefficients of variation (CV in %) calculated from the CV values obtained for each strain with 6 replicates.
(XLSX)

S6 Table Esters dataset. (XLSX)

S7 Table Micro-oxygenation and grape must interaction dataset. (XLSX)

S8 Table Phenotypic data of 35 commercial strains in five grape juices (raw data). (XLSX)

S9 Table Phenotypic data of 35 commercial strains in five grape juices (centered reduced data). (XLSX)
