# Supplementary figures and images for: Wine yeast phenomics: A standardized fermentation method for assessing quantitative traits of Saccharomyces cerevisiae strains in enological conditions
Source: PLoS One. 2018 Jan 19;13(1):e0190094. doi: 10.1371/journal.pone.0190094 (PMC5774694; doi:10.1371/journal.pone.0190094)

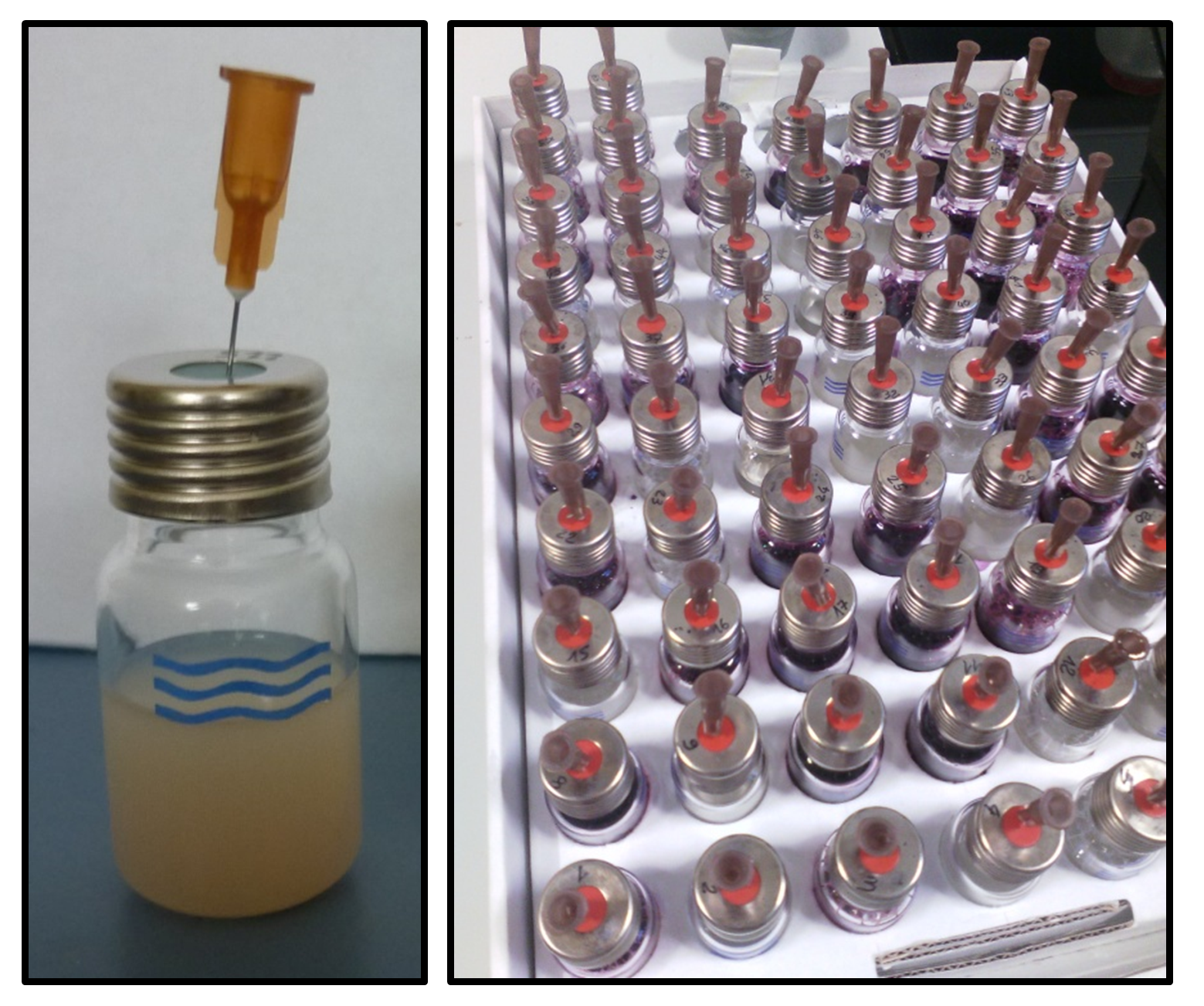

Supplement: S1 Fig — On the left, a SV filled with 5 mL of grape juice (SB14) and with a hypodermic needle to allow the CO2 release. On the right 70 vials on a rack illustrating the possibility of managing hundreds of fermentations in parallel. (TIF) [file pone.0190094.s001.tif]

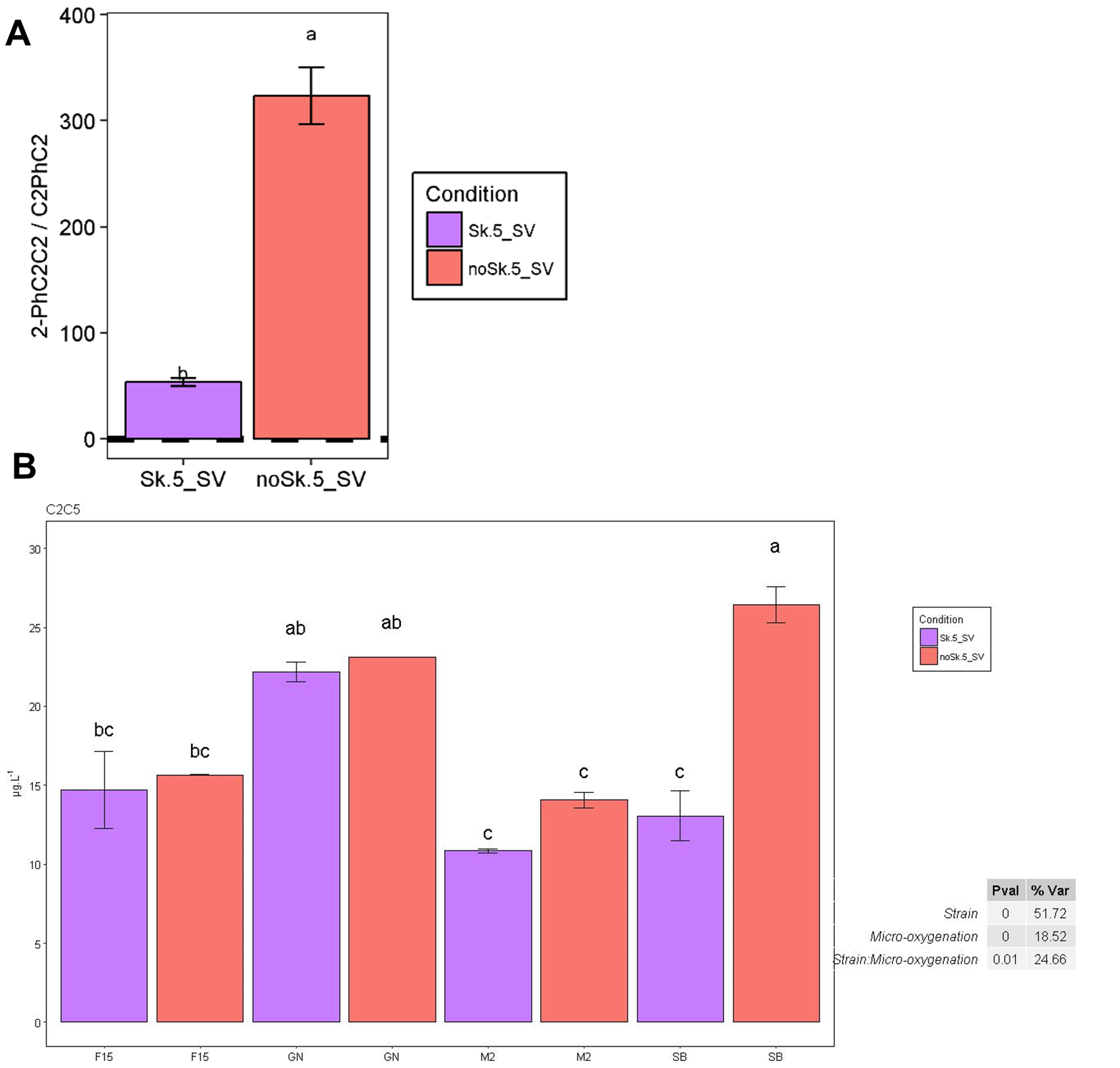

Supplement: S2 Fig — Panel A. The data shown are the mean proportion of PhC2C2 to C2PhC2 of the four strains in two replicates, the error bars represent the standard error. Different letters indicate significant differences between groups (Tukey’s honest significant difference test, significance level, α = 0.05). Panel B. The data shown are mean of two replicates, the error bars represent the standard error. Different letters indicate significant differences between groups (Tukey’s honest significant difference test, significance level, α = 0.05). Table represents ANOVA results (pval, and % of variance explained). (TIF) [file pone.0190094.s002.tif]

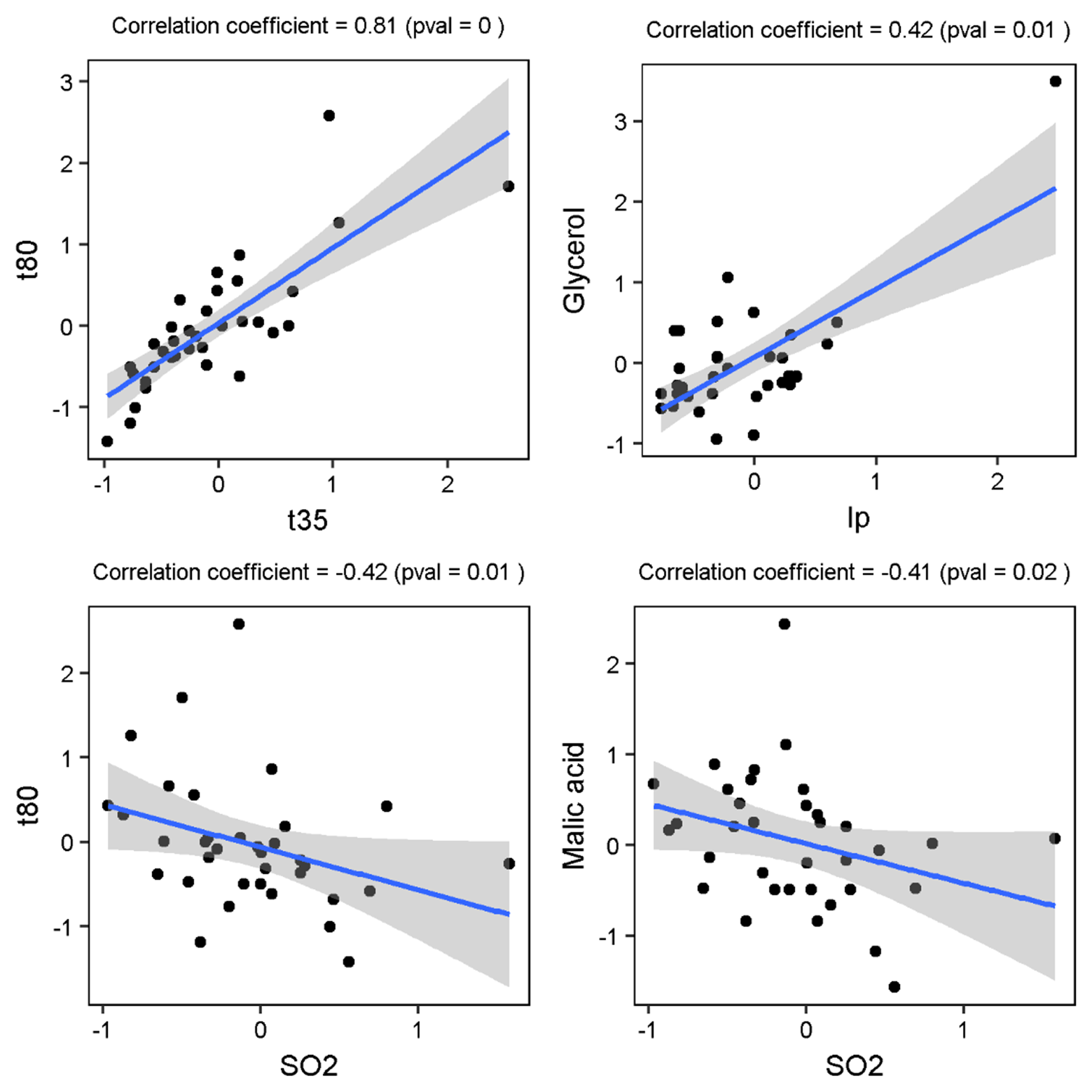

Supplement: S3 Fig — Scatter plots of correlated traits. Each dot represent the average phenotypic values of a strain across the five grape must from the normalized dataset. The blue line represents the linear regression line and the shaded area represents the confidence interval of the regression (0.95). (TIF) [file pone.0190094.s003.tif]

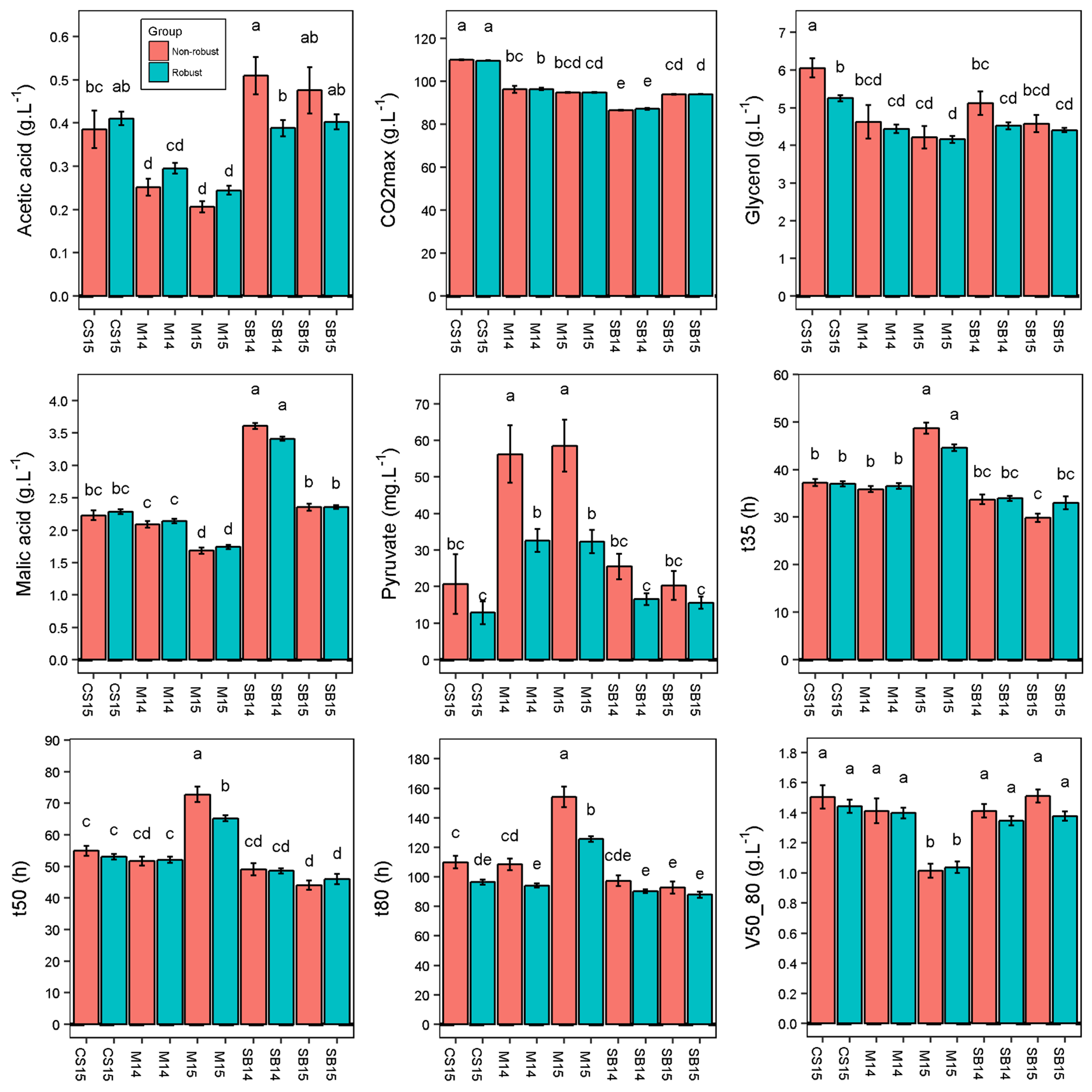

Supplement: S4 Fig — The data shown are the mean of eight strains (non-robust group) or 27 strains (robust group), the error bars represent the standard error. Different letters indicate significant differences between groups (Tukey’s honest significant difference test, significance level, α = 0.05). (TIF) [file pone.0190094.s004.tif]
